# Demand side management with electric vehicles and optimal renewable resources integration under system uncertainties

**DOI:** 10.1038/s41598-025-00752-6

**Published:** 2025-05-27

**Authors:** Mohamed Mostafa Eissa, Rania Abdel Wahed Swief, Tarek Saad Abdel Salam

**Affiliations:** Electrical Engineering Department, Faculty of Engineering, Ain Shams University, Cairo, Egypt

**Keywords:** Demand-side management, Distributed generators, Electric vehicle, Renewable energy resources, Zebra optimization algorithm, Energy grids and networks, Power distribution

## Abstract

The rapid growth of integrating electrical vehicles (EVs) into the distribution network has introduced complexities and power flow inefficiencies. To address these challenges, optimal renewable energy resources (RERs) integration along with applied demand-side management (DSM) contribute to managing load profiles and generation thus reducing costs. This should be smartly attained through selecting efficient optimization techniques to improve power quality, voltage profile, and reliability. This paper aims to investigate the effect of integrating EVs and applying peak load shifting (PLS) as a DSM strategy with the optimal allocation of distributed energy resources, specifically wind and photovoltaic (PV) systems, as distributed generators (DGs) on distribution networks. Taking into consideration the stochastic behavior of RERs, EVs demand elasticity of charging and discharging scenarios and load variance. The main objective of this work focuses on power loss reduction and implementing PLS to flatten the load profile and form a new loadability to reduce costs. The study is demonstrated on a typical IEEE 69-bus system, considering the load, EVs, and RERs profiles during weekdays in winter and summer seasons. The study examines the optimal size and location of combining two DGs (wind and PV), in addition to incorporating bidirectional plug-in hybrid electric vehicles into the system. The study utilizes the Zebra optimization algorithm (ZOA), in comparison with the Whale optimization algorithm (WOA), Grey wolf optimization algorithm (GWO), and Genetic algorithm (GA). The latter is employed only as a reference for comparison. For each season, the simulation is divided into two parts, each part consists of four cases. Part (1) is simulated assuming constant power integration for the RERs while part (2) considers their stochastic behavior. Also, optimal charging strategies for EVs are examined for cost-effectiveness during high penetration levels for the IEEE 123-bus system. The results demonstrated the effectiveness of the proposed algorithm in reducing power loss. Moreover, shifting peak hours flattens the load profile, thereby reducing costs and power loss across the distribution network. Furthermore, the performance of the ZOA dominates the WOA, GWO, and GA.

## Introduction

With the current consumers’ behavior and weather conditions, continuous fluctuations in electricity demand make it challenging to maintain an efficient and stable operation of power systems. These implications complicate the planning and operation of power grids, leading to increased costs, system losses, and instabilities. Additionally, the supply-demand balance has become more complex with the latter increase in renewable energy resources’ penetration^1^. Incorporating PV and wind as renewable energy resources (RERs) is widely utilized in several studies to improve grid efficiency due to their environmental benefits^2,3^.

Simultaneously, employing distributed energy resources (DERs) has a significant impact on improving the system performance^4^. Their optimal allocation reduces power loss and enhances the voltage profile of the distribution network^5^. However, the consequent uncertainties attributed to the unpredictable nature of RERs and load profile variability pose challenges to the optimization problem parameters, which was the trigger for cost allocation theories and strategies for saving costs^6^.

In recent years, the adoption of Electric vehicles (EVs) has served a critical role in the expanding demand for decarbonization and achieving zero carbon emissions^7^. Despite its economic benefit to the electricity market^8^, EVs impose harmonics to the network resulting from vehicles’ charging and discharging^9^. Contributing to the energy matrix, the random charging patterns of EV passengers make the behavior of EVs unpredictable and affect the short circuit level of the operating systems^10^. In conjunction with the reliability concerns of incorporating EVs into distribution systems^11^.

Among EVs, plug-in hybrid electric vehicles (PHEVs) consume an increasing share in the transportation sector. Many researchers studied their deployment on distribution networks and the corresponding increase in electric vehicle charging stations (EVCS) infrastructure^12^. Owing to its combined ability to transition between grid-to-vehicle (G2V) and vehicle-to-grid (V2G) modes. Adding up to the generation mix, integrating EVs and distributed generators (DGs) on the distribution network leverages the necessity of optimization to reduce losses and improve voltage profile^13^. Extensive research has been conducted on optimizing the bi-directional flow of the G2V and V2G methodologies for environmental benefits^14^. The majority focused on the V2G technology, emphasizing the enhancement of battery charging rates for improved lifespan and cost efficiency^15^.

In the same context, demand-side management (DSM) implementation plays a pivotal role in improving the efficiency and reliability of distribution networks^16^. Commonly in smart cities, applying DSM strategies motivates consumers to control their patterns of electricity usage during peak hours^17^. In previous literature, peak clipping and load scheduling were applied for energy conservation in distribution networks^18^. Also, demand response was deployed with EVs and DERs for grid stability^19^. Additionally, minimizing peak-to-average ratio (PAR) for DERs was employed on an IEEE 69-bus system^20^. While limited research has been conducted using peak load shifting^21^.

Furthermore, the need for optimal resource allocation is crucial in formulating the development of meta-heuristic optimization algorithms. Researchers optimized EV charging strategies to minimize the daily load peak and minimum peak-to-valley difference in load profiles^22^. Over the past years, several studies optimized the size and location of integrated multiple DGs and EVs separately on IEEE 33 radial distribution systems using whale optimization techniques (WOA) in the context of enhancing voltage stability index, power loss, and system stability^23^.

Other researchers used a multi-objective based on the V2G model by utilizing the Grey Wolf Optimization Algorithm (GWO) to find the optimal number of charging and discharging EVs for cost reduction^24^. Moreover, EV charging time and location of PV were optimized using a multi-particle swarm optimization algorithm (MPSO)^25^. Also, the impact of integrating DGs and EV was employed on an IEEE 33-bus system and the power loss reduction reached 70%^26,27^.

In the same framework, Genetic Algorithm (GA) has been deployed with EV integration and peak valley shifting for cost reduction. Results show that the optimized peak-to-valley load difference rate reached 58.9% of the distribution network^28^. While Competitive Swarm Optimizer (CSO) algorithm has been utilized on the IEEE 34-bus RDS system with EV integration to reduce costs by 1.34% of electricity expenses^29^. Also, the Arithmetic Optimization Algorithm (AOA) has been employed on IEEE 69-bus with the integration of EV only and peak load shifting to reduce costs^30^. Other researchers discussed the role of price incentives for EV users to optimize power quality^31^. While other researchers studied the impact of load patterns of EVs on power loss^32^.

One of the progressive recent optimization techniques is the Zebra Optimization Algorithm (ZOA). Inspired by the natural behavior of zebras in the wild and characterized by their unique behaviors of foraging and defense mechanisms against predators’ attacks ^33^. It has been recently employed with a new control approach using an intelligent hybrid controller to enhance the speed response, and maximum undershoots and overshoot for frequency controller in offshore fixed platform microgrids using tidal energy^34^. Also, it was used with controllers to improve the frequency performance of a hybrid interconnected dual-area of power grids and the dynamic behavior of automatic voltage regulator (AVR)^35–37^. Alternatively, it has been implemented with wireless sensor networks (WSNs) for node localization challenges^38^.

On the other hand, a few researchers studied the ZOA optimizer with the aim of power loss reduction and voltage stability index (VSI). Among these, ZOA was used to optimize the position and capacity of single and multiple DGs on an IEEE 33-bus radial system^39^. The results demonstrated a reduction percentage of 94.25% and VSI of 0.69643 to 0.9605. Besides, ZOA was utilized with the IEEE 14-bus and 30-bus systems to minimize costs, power losses, and voltage improvement^40^. Moreover, the robustness of the ZOA was demonstrated solely with EV integration against load and renewable energy fluctuations, to improve frequency stability with combined virtual inertia control (VIC) and fractional order PID (FOPID) controller in microgrids. The study demonstrated the critical role of integrating EVs in enhancing frequency response only ^41^. Rarely have the studies investigated the integration of combined EV and DER for power loss reduction on large-scale networks^42^.

Based on the aforementioned review, there is still a critical need to address the impacts of combined integration of EVs and RERs on large-scale networks. Most studies optimized the size and location of connecting wind, and PV as separate DGs, but rarely investigated their connection as a hybrid with EVs integration in V2G mode. Also, many researchers defined single objective functions related to power loss and voltage profile enhancement. While the effect of setting multi-objective functions of power loss and peak load shifting is still undefined on large-scale networks in previous studies. Moreover, the impact of the seasonal stochastic behavior of EVs and DER with load uncertainties in respect to power loss is still not covered in detail by recent studies.

Although previous studies often examined peak average ratio and peak load clipping as demand-side management strategies, however, few researchers applied peak load shifting in optimizing the DG locations combined with optimal charging strategies for power loss reduction. There is still a gap in addressing peak load shifting on large-scale networks such as IEEE 69-bus and IEEE 123-bus systems along with the combined integration of DERs and EV during load uncertainty. In addition, many studies explored the allocation of DGs using various optimization techniques but with a limitation to exploring ZOA on a more complex scale. Even though ZOA has proven its efficiency with power loss reduction, as far as the author reviewed, it has not been demonstrated with demand-side management strategies. Nevertheless, considering seasonal factors for DERs and EVs. Although ZOA has been utilized in comparison to various well-known benchmarks in numerous research recently. However, its implementation with the combined integration of EVs and DERs remains unexplored thoroughly, especially on large-scale networks.

To address these limitations, this paper aims to investigate the effect of EV integration and apply peak load shifting as a demand-side management strategy. Unlike previous studies that optimize DERs placement or DSM separately, this work simultaneously investigates both providing a comparative analysis of their impact on minimizing power losses and peak load shifting during uncertainties for cost reduction. The study is demonstrated primarily on a standard IEEE 69-bus to optimally allocate the size and location of two DERs considering their stochastic behavior during winter and summer on various case studies. The approach combined power loss reduction and peak load shifting to flatten the load profile and reduce costs. The proposed optimization technique to be explored in the solution is the ZOA, in comparison to other techniques. Secondly, the findings serve as an input for further investigation during high penetration levels of EV. Lastly, the system configuration is deployed on an IEEE 123-bus system to validate the outcomes on a larger scale. The main contributions of this study can be summarized as follows:

- Integrate EVs into the grid considering the elasticity of their charging (G2V) and discharging (V2G) scenarios during the winter and summer seasons.
- Apply DSM through peak load shifting during uncertainties thus enhancing the load profile for cost reduction and improved load factor.
- Utilize a multi-objective function for power loss reduction and peak load shifting to improve a typical IEEE 69-bus performance.
- Allocate two distributed energy resources (wind and PV) as a hybrid and incorporate EVs considering their stochastic behavior during winter and summer seasons along with load uncertainty.
- Apply the Zebra Optimization technique and compare its performance to GWO, WOA, and GA in achieving maximum power loss reduction.
- Investigate the impact of different penetration levels of EVs on distribution networks for cost reduction.
- Explore the effectiveness of the proposed model on an IEEE 123-bus system.

The paper structure is organized into seven sections. Starting with the introduction and literature survey represented in “Introduction” section. Problem formulation and objective functions are explained in “Problem formulation” section. The system model under investigation featuring the wind, PV, and EV integration profiles, and demand-side management strategy used is demonstrated in “System under study” section. In “Proposed optimization technique” section, the Zebra Optimization Algorithm (ZOA) is illustrated with the flow chart and pseudo-code. Simulation analysis and results are portrayed in “Simulation results” section. Finally, the result’s discussion and conclusions are given in “Discussion” and “Conclusion” sections, respectively.

## Problem formulation

Finding the optimal configuration of DG placement and load distribution is the most important goal for power loss reduction and cost reduction. Along with effective demand-side management, reducing the peak demand enhances grid stability. The main objective of this paper is to search the optimal size and location of integrating two DGs (hybrid) with EV integration on a typical IEEE 69-bus system to minimize power losses and apply demand-side management through peak load shifting to flatten the load profile for cost reduction and fixed operation.

The multi-objective function (MOF) for searching the optimal solution can be described by (1):

1 $$MOF = \left( {F1,\;F2} \right)$$

where *F1* is set to minimize the total active power loss as the first objective function and *F2* is set to shift the peak load hours to flatten the profile as the second objective and create a new loadability^20^.

### Probabilistic power loss

The probabilistic power loss formulated for the DGs by calculating the mean of the hourly active power. The total active power loss is calculated using (2) and (3):

2 $${P}_{DG loss}= \sum_{i=1}^{n}{I}^{2}*(R)$$

3 $${P}_{loss}=mean( \sum_{1}^{N}{P}_{DG loss})$$

where *P*_*DG loss*_ represents the DG hourly active power loss, *I* is the current passing through the section, *R* is the section resistance, *n* shows the number of sections and *N* is the number of hours.

### Peak load shifting

In the context of demand-side management, peak load shifting is applied to reduce costs around peak hours. Shifting to off-peak hours takes place when the load power percentage exceeds the (mean + 0.5 × Std)^20^. The peak load shifting is implemented using (4), (5) and (6):

4 $$Maximum = mean + 0.5 \times Std$$

5 $$Minimum = mean - 0.5 \, \times \, Std$$

6 $$Diff = load\_power\;\left( {peak\;hours} \right) - Maximum$$

where *Std* represents the standard deviation, *mean* is the average power and *Diff* is the difference between the load power at peak hours and *Maximum*.

The load power across the 24 h is categorized as peak and off-peak periods. The load power exceeding *Maximum* is identified as peak periods that shall be shifted, while the load power below *Minimum* is identified as off-peak periods for valley filling. The difference between the load power at peak periods and *Maximum* is calculated. Then, the total *Diff* represents the excess demand that needs to be rescheduled. This excess demand shall be shifted from periods of high demand (peak hours) to periods of low demand (off-peak hours), thus reducing peaks. A new loadability profile is generated to flatten the profile during the day and reduce costs, which can be described in (7) and (8):

7 $$New\;loadability = load\_power\;\left( {peak\_hours} \right) - Diff$$

8 $$New\;loadability = load\_power\;\left( {off\_peak\_hours} \right) + Diff$$

### System under study

**Fig. 1:**
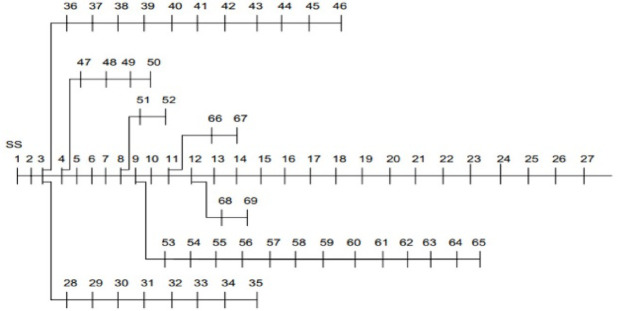
A Standard IEEE 69-bus distribution network.

In this research, a standard IEEE 69-bus distribution network, as shown in Fig. 1, is examined to study the impact of incorporating RERs and EVs. The distribution network consists of 69 nodes and 7 lateral feeders. The line and bus data of the IEEE 69-bus system is represented in Table A1 in the Appendix. The investigation employs the backward-forward sweep method, and optimization algorithms are applied to assess the outcomes. The assumed level of penetration for renewable resources is 20% of the total active power 3801.89 kW and reactive power 2694.1 kVAR at an operating voltage of 12.66 kV. The primary goal is to minimize power losses, and the secondary goal is to implement peak load shifting through the integration of these resources as DGs. The proposed shifting methodology controls at each hour the un-shiftable load by setting a certain threshold using the load profile’s mean and standard deviation to identify the overloaded consumption at peak hours and shifted peak clipping, to the underloaded hours at off-peak hours by valley filling. The analysis has been conducted using MATLAB software on a 1.80 GHz intel core i7-10510U CPU with 8 GB of RAM.

### Load profile

The variation in load profile is introduced using the IEEE Reliability Test System (RTS)^43^ which represents the hourly peak load as a percentage of the daily peak load during weekdays in winter and summer seasons as shown in Table 1.

**Table 1:** Hourly peak load in percent of daily peak (a) Winter season, (b) Summer season.

(a) Winter season												
Hours	1	2	3	4	5	6	7	8	9	10	11	12
% Load	0.67	0.63	0.60	0.59	0.59	0.60	0.74	0.86	0.95	0.96	0.96	0.95
Hours	13	14	15	16	17	18	19	20	21	22	23	24
% Load	0.95	0.95	0.93	0.94	0.99	1	1	0.96	0.91	0.83	0.73	0.63

(b) Summer season												
Hours	1	2	3	4	5	6	7	8	9	10	11	12
% Load	0.64	0.6	0.58	0.56	0.56	0.58	0.64	0.76	0.78	0.95	0.99	1
Hours	13	14	15	16	17	18	19	20	21	22	23	24
% Load	0.99	1	1	0.97	0.96	0.96	0.93	0.92	0.92	0.93	0.87	0.72

### Wind and PV integration

**Fig. 2:**
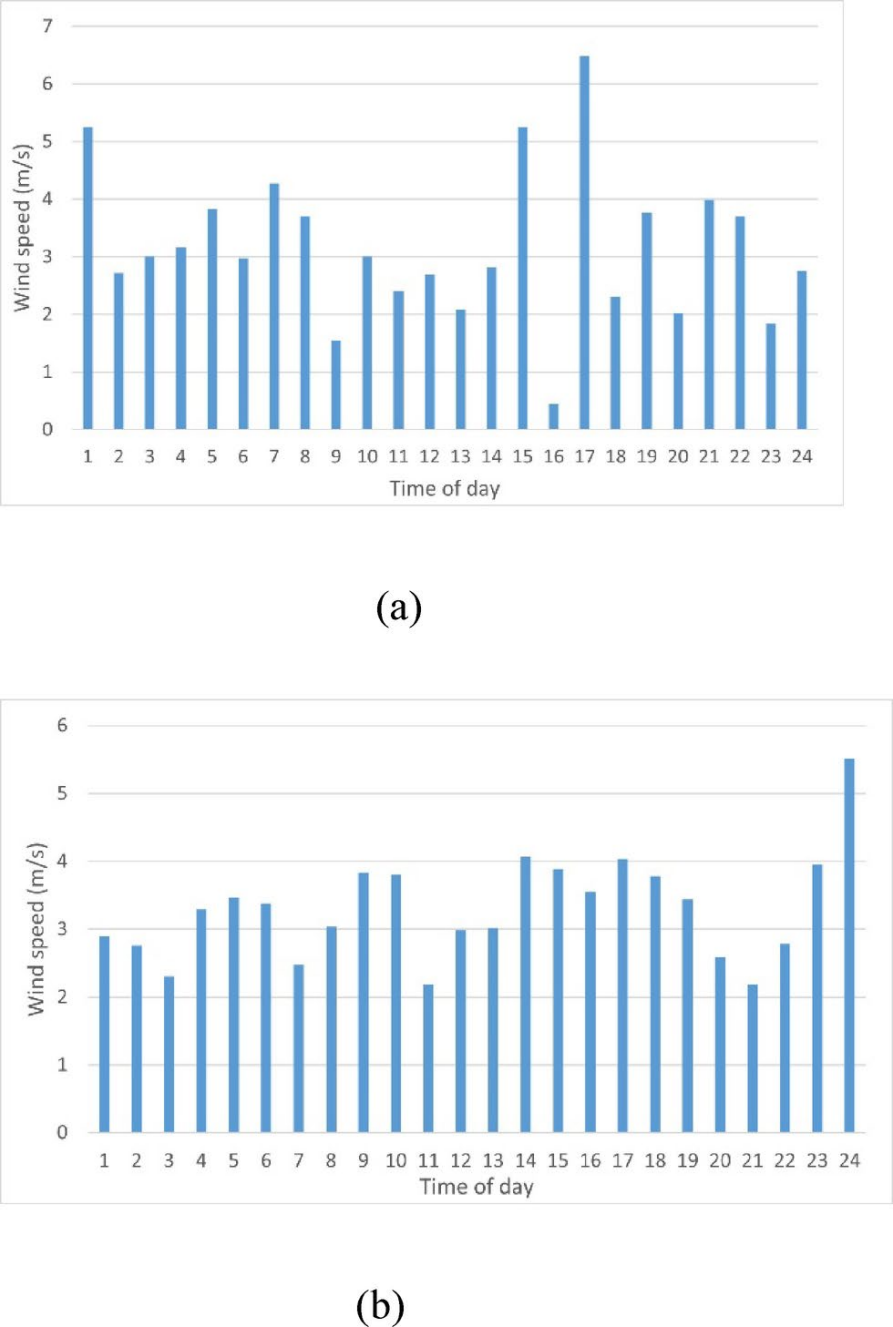
Wind speed hourly forecast during the day (**a**) Winter season, (**b**) Summer season.

**Fig. 3:**
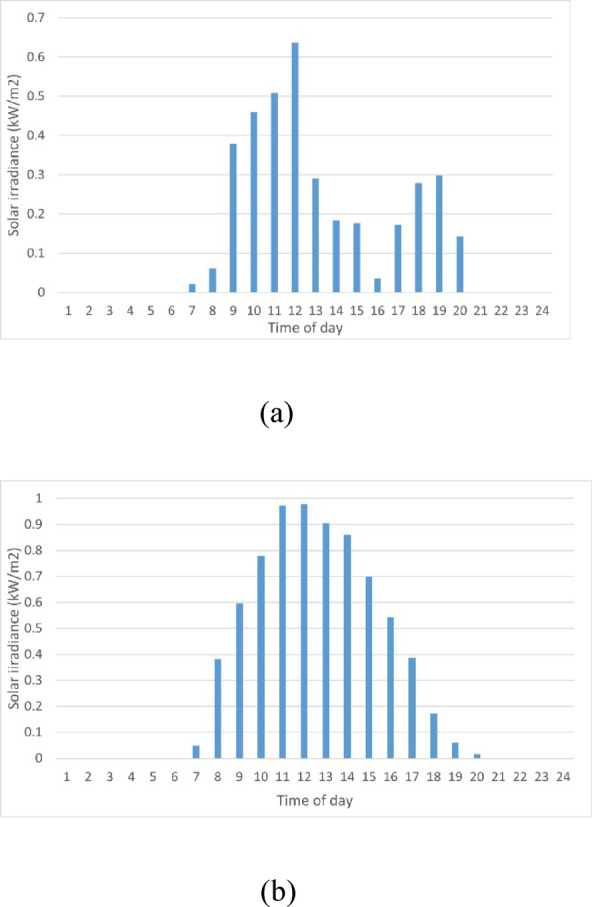
Solar irradiance hourly forecast during the day (**a**) Winter season, (**b**) Summer season.

Renewable energy resources have become key components in the global shift towards decarbonization and sustainability in distribution networks. Their environmental and economic benefits make them appealing to researchers. Since solar irradiance and wind speed are influenced by many random factors such as weather conditions and geographical location, they should be deployed considering their stochastic behavior during uncertainties^44^. In this study, the probabilistic active power of wind and PV is introduced by using the probability density function (PDF) of each. The real data of wind speed and solar irradiance profile during the day in winter and summer seasons^45^ is shown in Figs. 2 and 3, respectively. The temperature ranges fall between 5.5/11.1 °C (highest reading) and −10/−4.4 °C (lowest reading) during winter season, and 28.8/23.3 °C (highest reading) and 12.8/7.2 °C (lowest reading) during summer season. To enhance the stability of the system under study, it is assumed that wind and PV sources are integrated with a level of penetration of 60% and 40%, respectively, of the total renewable generation capacity.

### Electric vehicle integration

The massive integration ofPHEVs into power systems recently offers significant potential for enhancing the network’s performance. Yet, drivers’ stochastic charging and discharging behavior affect system stability and reliability. Moreover, the forecasted demand for EVs is sensitively affected by seasonal factors owing to changes in driving habits and charging strategies on the network.

The EV integration is estimated to possess two scenarios. In the first scenario, the EV is presumed to be integrated from 7:00 pm to 7:00 am (12 h). During which, most EV drivers are back from work and begin charging their vehicles. This G2V phase considers EV consumption as a load. In the second scenario, the EV integration is considered from 7:00 am to 7:00 pm (12 hours). Throughout this, most EV drivers are working or running errands and could make use of this V2G phase, thus the EV consumption is deemed as a distributed generator (DG).

**Fig. 4:**
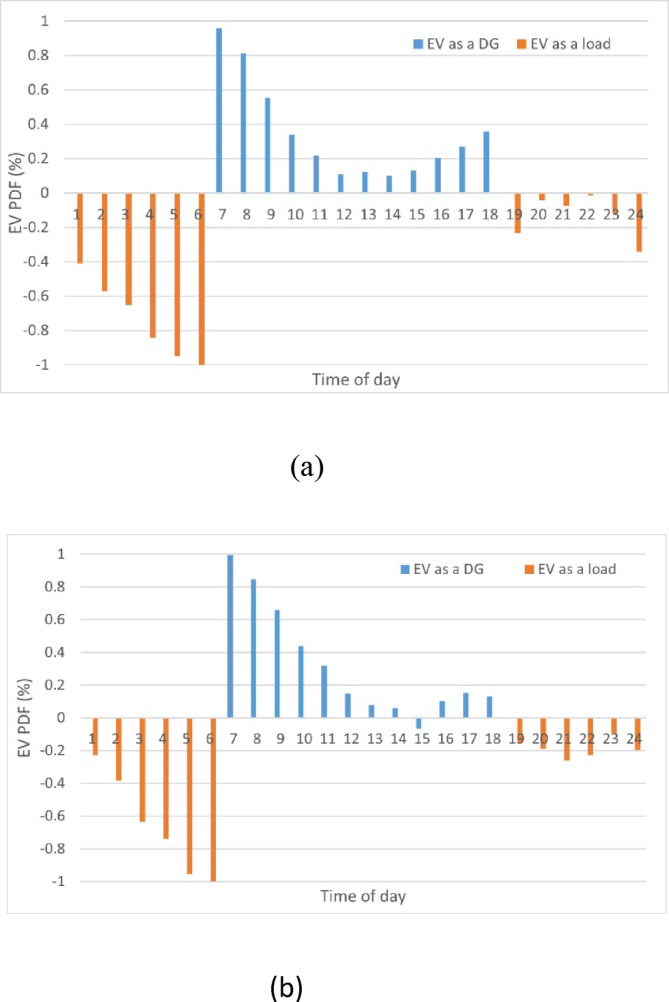
Electric Vehicle PDF (**a**) Winter season, (**b**) Summer season.

In this study, only 10% of the total number of consumers in the distribution network^46^ assumed to own an EV with an average battery consumption of 70 kWh^47^. Under a constraint that the EV battery state-of-charge (SOC) exceeds 50%, to ensure sufficient reserves during the return journey. Moreover, during the analysis, the maximum EV battery capacity utilized on the grid in the V2G mode is estimated to be 20%, to avoid the risk of depleting their charge. The drivers’ behavior is not constant during the day and driving patterns change over the hour. The uncertainties of EV are managed by incorporating the hourly profile of EV consumption to ensure that the results are more practical. Also, the EV is integrated using the PDF of forecasted processed real data of EV consumption on the electricity grid of Spain^48^ using the ANFIS optimizer in winter and summer seasons over 24 h^49^, as shown in Fig. 4. The average temperature ranges fall between 16/6 °C (highest reading) and 10/3°C (lowest reading) during winter season, and 32/19 °C (highest reading) and 26/15 °C (lowest reading) during summer season.

### Peak load shifting

One of the key aspects of demand-side management is peak load shifting which involves incentivizing consumers to shift their energy consumption away from peak demand periods to off-peak times through pricing schemes, incentives, and automated systems^50^. This shall alleviate stress on the grid during peak periods, improve the overall system efficiency, and reduce the need for costly infrastructure. In this study, the peak load shifting objective is implemented using the mean and standard deviation, as illustrated earlier, on the IEEE 69 bus system considering the load uncertainty by the IEEE RTS, with a focus on the hourly load profile of bus number 61.

A trial was simulated using the mean and standard deviation into shifting the load power for the peak and off-peak hours using the following steps:

- Calculate the mean load across the 24 h and *Std.*
- Compute *Maximum* and *Minimum* using (4) and (5).
- Identify peak and off-peak periods
- Determine the *Diff* at each peak period using (6).
- Evenly distribute the total *Diff* from peak hours across multiple off-peak hours using (7) and (8).

The calculation analysis is detailed in Table A2 in the Appendix. The table represents a reduction in total load demand from 24,780 kW to 24,535 kW, and the load factor slightly improved from 0.83 to 0.8736. Even though the total Diff determined to be shifted reached 796 kW, the new profile is partially flattened after valley filling with a peak-to-average ratio decreasing from 1.2 to only 1.14.

**Fig. 5:**
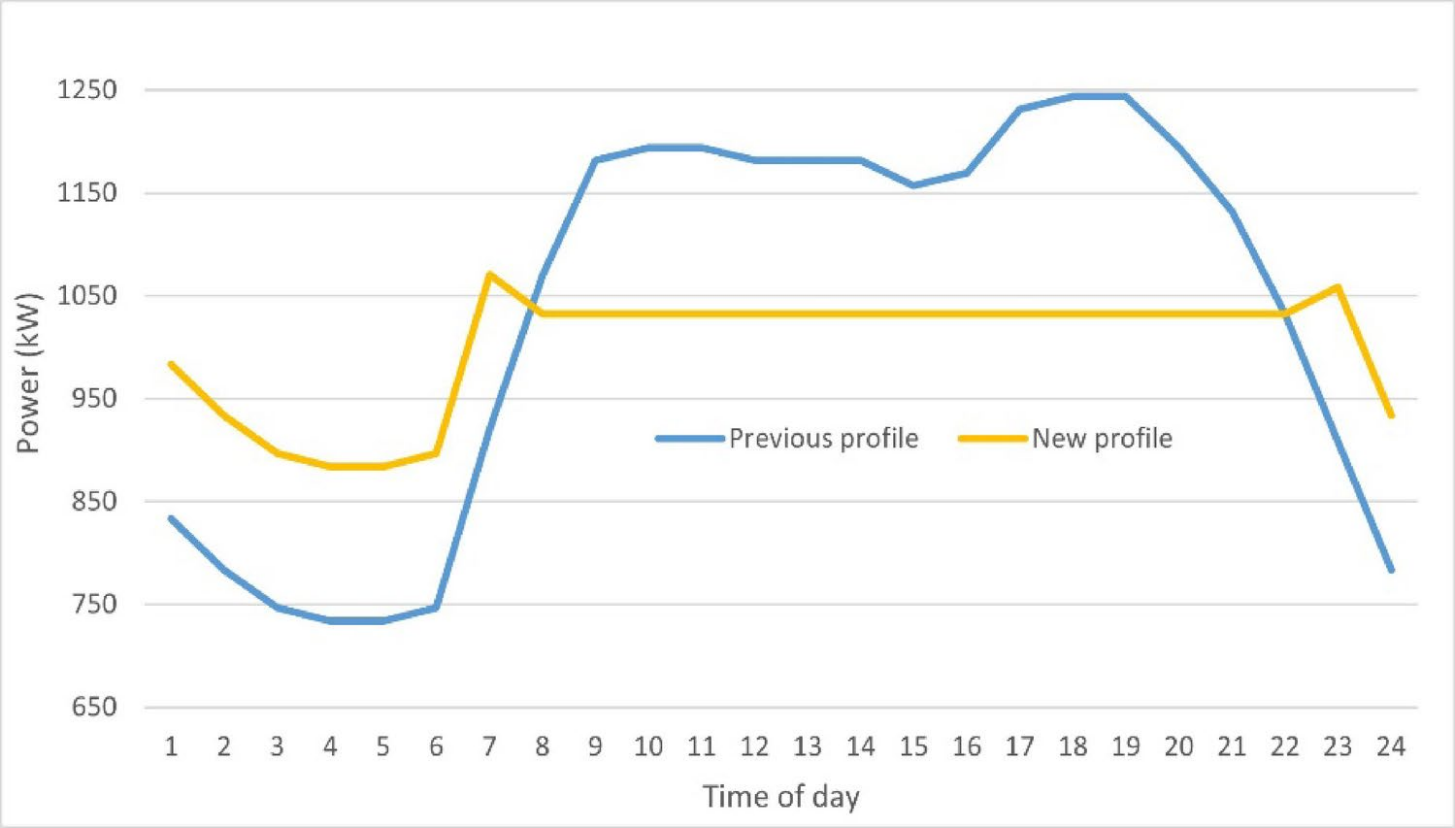
Bus number 61 previous and new loadability profile using mean only.

A second approach is applied by calculating the *mean* value only using the previous steps to ensure a smoother load profile. Table A3 in the Appendix illustrates the detailed calculation analysis. The total excess demand in this case reached 2102 kW, which shall be utilized to be shifted. The total load demand across the 24 h is reduced from 24,780 kW to 24,030 kW. Figure 5 represents the previous and new load profile for bus number 61 after peak load shifting throughout the day using the mean only.

The figure reveals that the peak demand hour before applying peak load shifting is from (18) to (19) p.m. while the off-peak load demand hour is from (4) to (5) a.m. Also, the figure shows a significant improvement in the total daily load profile after the implementation of the shifting strategy, where the total demand at full load for bus number 61 flattened from 1244 to 1032.52 kW. Moreover, the load factor, which is calculated as the average of the 24 h load demand divided by the peak load demand within the 24 hours, has improved from 0.83 to 0.935. In addition, the peak-to-average ratio using the mean only approach is reduced to 1.069, compared to 1.14 using the mean and standard deviation. Accordingly, the mean only model is chosen to proceed upon incorporating the system under study which gives a more promising values to flatten the curve, reduce system losses and improve performance. As demonstrated, the demand response contributes to peak reduction and improvement to the load factor, thus system efficiency. Also, shifting the peak load hours flattens the load profile and forms a new loadability, which shall be examined further in the case studies.

### Proposed optimization technique

In this section, the proposed optimization technique is the nature-inspired Zebra Optimization Algorithm (ZOA). distinguished by their foraging behavior where pioneer zebras lead the way for other zebras into the forage. Their defense strategy is using a zigzag pattern to escape predators, and this sometimes confuses the predator. The ZOA algorithm^34^ consists of three phases:

#### Initialization

ZOA is considered a population-based optimizer of which zebras are members. Each zebra can be identified as a candidate solution within the problem’s search space where its position determines the value of the decision variable. Hence, the population of zebras can be modeled using a matrix while each zebra can be modeled using a vector and the values of the problem variables are the elements of this vector. The population matrix for ZOA can be represented by using (9):

9 $${\text{X}} = \left[ {\begin{array}{*{20}c} {X_{1} } \\ . \\ . \\ . \\ {X_{i} } \\ . \\ . \\ . \\ {X_{N} } \\ \end{array} } \right]_{{N{ } \times { }m}} = { }\left[ {\begin{array}{*{20}c} {x_{1,1} } & \cdots & {x_{1,j} } & \cdots & {x_{1,m} } \\ \vdots & \ddots & \vdots & \ddots & \vdots \\ {x_{i,1} } & \cdots & {x_{i,j} } & \cdots & {x_{i,m} } \\ \vdots & \ddots & \vdots & \ddots & \vdots \\ {x_{N,1} } & \cdots & {x_{N,j} } & \cdots & {x_{N,m} } \\ \end{array} } \right]_{{N{ } \times { }m}}$$

where *X* is the zebra population, *Xi* is the ith zebra, *Xi,j* is the value for the jth problem variable by the ith zebra, *N* is the number of zebras (population members), and *M* is the number of decision variables.

Based on the proposed value for the problem variables of each zebra, the objective function can be evaluated. Each zebra denotes a candidate solution to the optimization problem. Thus, the obtained values for the objective function can be presented as a vector using the matrix (10):

10 $${\text{F}} = \left[ {\begin{array}{*{20}c} {F_{1} } \\ . \\ . \\ . \\ {F_{i} } \\ . \\ . \\ . \\ {F_{N} } \\ \end{array} } \right]_{{N{ } \times { }1}} = { }\left[ {\begin{array}{*{20}c} {F(X_{1} )} \\ . \\ . \\ . \\ {F(X_{i} )} \\ . \\ . \\ . \\ {F(X_{N} )} \\ \end{array} } \right]_{{N{ } \times { }1}}$$

where, *F* is the vector of objective function values and *Fi* is the objective function value obtained for the ith zebra.

By comparing the obtained values, the quality of the corresponding candidates can be analyzed and the best candidate solution for the problem can be identified. For each iteration, the position and the values of the objective function are updated and consequently, the best candidate solution is identified.

In the wild, there are two natural behaviors for zebras that have been used to update the members of the ZOA population in two different phases for each iteration. The first phase is foraging behavior while the other phase is defense strategies against predators.

#### Foraging behavior (phase I)

Mainly the diet of zebras is grasses and sedges. By updating the population members based on the simulations of zebra’s behavior while exploring for forage. Plain zebras are pioneer grazers that devour the canopy of upper and less nutritious grass and provide other species that need shorter and more nutritious grasses below. Pioneer zebras are the best population members that lead other members toward their position in the search space. The foraging phase and updating the position of zebras can be modeled using (11) and (12):

11 $$x_{i,j}^{{new,{ }P1}} = {\text{ xi}},{\text{j}} + {\text{r }}.\left( {{\text{PZj }}{-}{\text{I }}.{\text{ xi}},{\text{j}}} \right)$$

12 $${\text{Xi }} = \left\{ {\begin{array}{*{20}c} {X_{i}^{{new,{ }P1}} ,} & { F_{i}^{{new,{ }P1}} < F_{i} ;} \\ {X_{i} , } & {else,} \\ \end{array} } \right.$$

where ${X}_{i}^{new, P1}$ is the new status of the ith zebra based on phase I, ${x}_{i,j}^{new, P1}$ is the jth dimension value, ${F}_{i}^{new, P1}$ is the objective function value, *PZ* is the pioneer zebra (best member) while *PZj* is the jth dimension, *r* is a random number in the interval [0,1], *I* is the round (1 + rand) where I €{1,2} and *rand* is a random number in the interval [0,1].

#### Defense strategies against predators (phase II)

By updating the position of the population members based on the simulations of the zebra’s defensive strategy against predators’ attacks. Mainly, lions are predators however, zebras are also threatened by dogs, hyenas, cheetahs, leopards, and crocodiles. The defense strategy of zebras depends on the predator. While being attacked by lions, they escape in zigzag patterns and sideways random movements. On the other hand, zebras are even more aggressive when being attacked by small predators. If one of the two conditions occurs with the same probability:

i. Escape strategy when being attacked by lions.
ii. Offensive strategy when being attacked by other predators.

These two strategies can be represented in (13) and (14):

13 $$x_{i,j}^{new,P2} = \left\{ {\begin{array}{*{20}c} {S_{1} : x_{i,j} + R.\left( {2r - 1} \right)} \\ {.\left( {1 - \frac{t}{T}} \right).x_{i,j} , \;P_{s} \le 0.5 } \\ {S_{2} : x_{i,j} + r.\left( {AZ_{j} - I.x_{i.j} } \right),\quad \quad else} \\ \end{array} { }} \right.$$

14 $${\text{Xi }} = \left\{ {\begin{array}{*{20}c} {X_{i}^{{new,{ }P2}} ,} & {F_{i}^{{new,{ }P2}} < F_{i} ;} \\ {X_{i} ,} & {else,} \\ \end{array} } \right.$$

where *S1* is the escape strategy mode, *S2* is the offensive strategy mode, ${X}_{i}^{new, P2}$ is the new status of the ith zebra (phase II), ${x}_{i,j}^{new,P2}$ is the jth dimension value, ${F}_{i}^{new, P2}$ is the objective function value, *t* is the iteration contour, *T* is the maximum number of iterations, *R* is the constant number equal to 0.01, *Ps* is the probability of choosing one of two strategies [0,1], *AZ* is the status of attacked zebra and *AZj* is the jth dimension value.

**Fig. 6:**
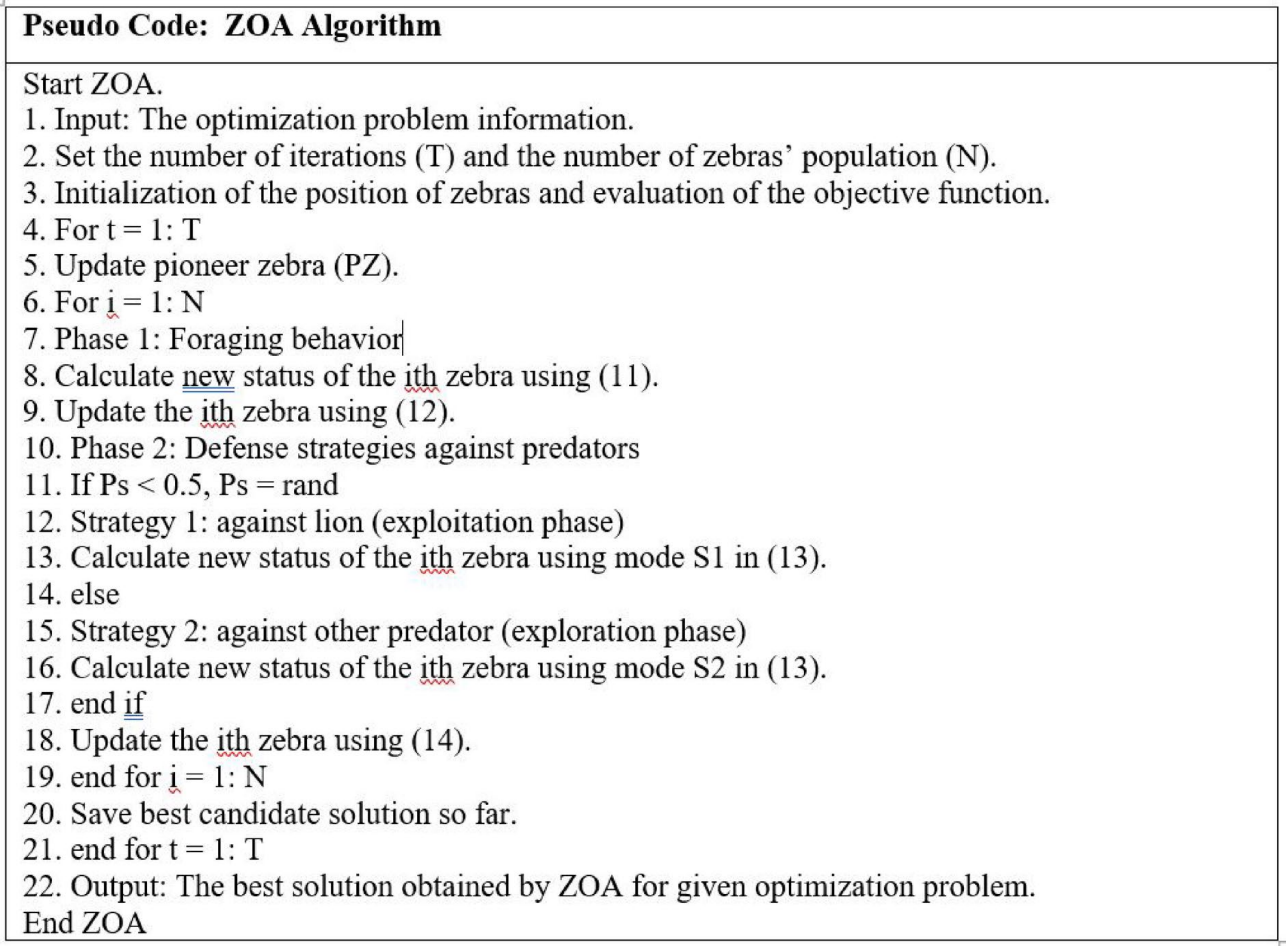
ZOA pseudo code.

**Fig. 7:**
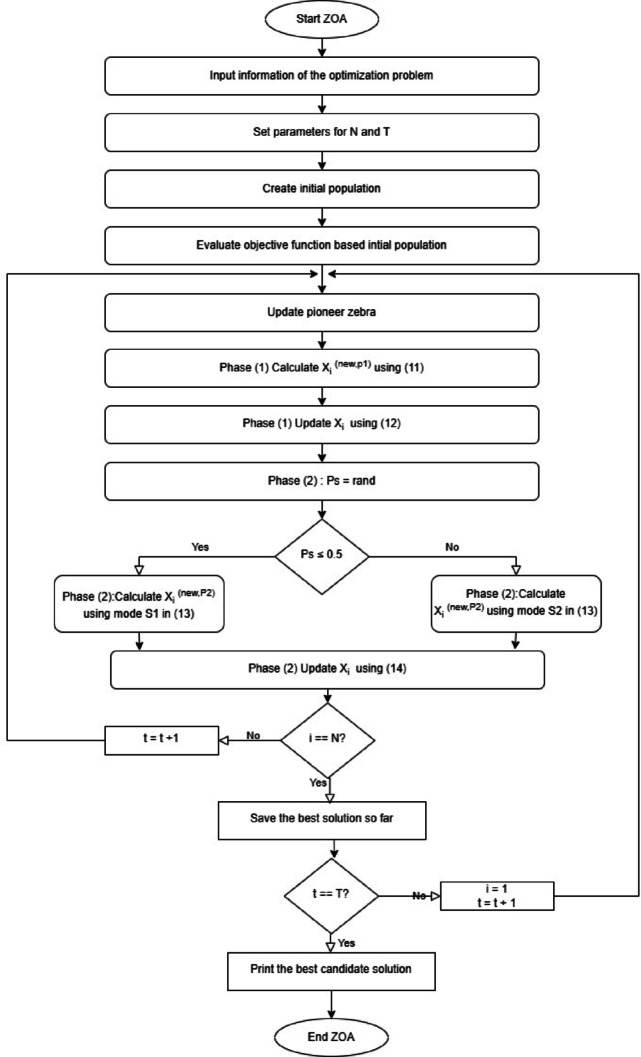
ZOA flowchart.

The ZOA steps can be expressed in the pseudo-code in Fig. 6:

Moreover, the ZOA flow chart can be represented as shown in Fig. 7:

### Simulation and results

The study aims to investigate the impact of the increased adoption of EVs and RERs on the performance of power grids. Their combined impact poses challenges regarding power loss that necessitates careful analysis. The proposed investigation employed an IEEE 69-bus radial distribution system over eight cases for the winter and summer seasons, separately. The EV charging patterns and DER behaviors are modeled by their respective contribution of the injected active and reactive power to the grid. The level of renewable energy integration was set to be 20% of the total integrated active and reactive power of the IEEE 69-bus system. Firstly, the winter season data is introduced for the simulation to be evaluated. The simulation cases under analysis are divided into two parts as in Table 2, to demonstrate the significance of the investigation. Initially, a simulation for the backward-forward sweep is implemented on the system as the baseline case without integrating any DGs or EVs. The total power loss and voltage index are 224.96 kW and 0.9734, respectively.

**Table 2:** Simulation cases study.

Part (1): Fixed Integration of DGs	Part (2): Variable Integration of DGs
Case (A): considering 100% loading	Case (E): considering 100% loading
Case (B): considering load variance with peak load shifting	Case (F): considering load variance with peak load shifting
Case (C): integrating EV considering 100% loading	Case (G): integrating EV considering load variance
Case (D): integrating EV considering 100% loading with peak load shifting	Case (H): integrating EV considering load variance with peak load shifting

Each part consists of four cases. Part (1) comprises cases from (A) to (D) examining the impact of fixed integration of DGs while part (2) comprises cases from (E) to (H) exploring the effect of DG integration under uncertainties. Cases (A) and (E) investigate the impact of considering a full loading day, while in cases (B) and (F) the variability in load profile is considered after peak load shifting. The integration of EV outcomes is examined in cases (C) and (G). Finally, in cases (D) and (H), the peak load shifting is considered with EV integration. In each case, the optimization techniques are applied to obtain the optimal allocation and size of each unit to minimize power losses.

In part (1), the PV and wind were integrated as two DGs (hybrid) assuming a constant power output with a level of integration of 60% and 40%, respectively, of the total renewable generation capacity. This part comprises cases from (A) to (D). The simulation was utilized by using the ZOA and compared with WOA, GWO, and GA for 50 iterations.

#### Case (A): Fixed integration of wind/PV power with 100% loading

In this case, the system was simulated considering full load hours throughout the day without applying peak load shifting. The wind/PV are integrated with a constant power output regardless of their stochastic behavior. The total probabilistic power loss was identified as an objective, and the results are represented in Table 3.

**Table 3:** Simulation case (A) results.

Applied algorithm	Optimal location (1) PV	Optimal location (2) Wind	DG PV size (KW)	DG wind size (KW)	Power loss (KW)	Reduction %
Base case	–	–	–	–	224	–
ZOA	61	64	433.33	303.96	87.78	60.81
WOA	65	63	30.04	245.84	145.23	35.17
GWO	64	27	432.38	219.14	133.58	40.37
GA	64	62	456	247.7	98.4	56.07

The results show that the maximum power loss reduction reached is 60.81% by utilizing the ZOA. The power loss reached 87.78 kW by optimally using 433.33 kW PV modules and 303.96 kW of wind modules at the placement of PV and wind at bus numbers 61 and 64 respectively. On the other hand, WOA, GWO, and GA power loss reached 145.23 kW, 133.58 kW, and 98.4 kW respectively. Even though GA performance was better than WOA and GWO in achieving the maximum power loss reduction, however, it required the longest time during iterations.

#### Case (B): Fixed integration of wind/PV power and load variance after peak load shifting

While in this case, the system was simulated considering the variable loading hourly percentage using RTS. Also, the integration of wind/PV is considered as constant power regardless of their stochastic behavior. The total probabilistic power loss and peak load shifting were identified as objective functions, and the results are represented in Table 4.

**Table 4:** Simulation case (B) results.

Applied Algorithm	Optimal location (1) PV	Optimal location (2) Wind	DG PV size (KW)	DG Wind size (KW)	Power loss (KW)	Reduction %
Base case	–	–	–	–	224	–
ZOA	61	63	455.14	292.66	32.79	85.36
WOA	62	65	439.18	100.14	49.62	77.85
GWO	61	21	456	268.3	59.86	73.28
GA	63	61	424.01	193.05	47.6	78.75

The results reveal a significant reduction in the power loss after implementing peak load shifting in comparison to case (A). Also, the maximum reduction in power loss is achieved by utilizing the ZOA followed by GA, WOA, and GWO with corresponding reduction percentages of 85.36%, 78.75%, 77.85%, and 73.28%, respectively. By applying the ZOA, the optimal placement of PV and wind modules is at bus numbers 61 and 63 with a capacity of 455.14 kW and 292.66 kW, respectively.

#### Case (C): Fixed integration of wind/PV power with EV and 100% loading

With respect to this case, the integration of EV is examined on the proposed model without applying peak load shifting. The system was simulated considering fixed integration of constant wind/PV powers and full load hours throughout the day. Subsequently, the EV is integrated using the PDF of the forecasted processed data of EV consumption in winter over 24 h. The total probabilistic power loss was identified as an objective function. The results are represented in Table 5.

**Table 5:** Simulation case (C) results.

Applied algorithm	Optimal location (1) PV	Optimal location (2) Wind	DG PV size (KW)	DG Wind size (KW)	Power loss (KW)	Reduction %
Base case	–	–	–	–	224	–
ZOA	61	64	435.92	293.18	78.82	64.81
WOA	63	65	438.97	292.83	80.15	64.22
GWO	64	62	442.93	298.31	82.12	63.34
GA	64	61	346.15	197.03	104.89	53.17

As indicated from the results, the EV integration considering the two scenarios of V2G and G2V along with DERs increases power loss reduction notably in comparison to case (A). In addition, the maximum power loss reduction is achieved using ZOA, followed closely by WOA, GWO, and GA with a reduction percentage of 64.81%, 64.22%, 63.34%, and 53.17%, respectively. Moreover, the ideal placement of 435.92 kW PV modules and 293.18 kW wind modules is at bus numbers 61 and 64 respectively.

#### Case (D): Fixed integration of wind/PV power with EV and load variance along with peak load shifting

Given this case, the system was simulated considering the variable loading hourly percentage using RTS along with fixed integration of wind/PV powers. Subsequently, the EV is integrated using the PDF of forecasted processed data of EV consumption in winter over 24 h. The total probabilistic power loss was identified as an objective function with applying peak load shifting. The results are represented in Table 6.

**Table 6:** Simulation case (D) results.

Applied algorithm	Optimal location (1) PV	Optimal location (2) Wind	DG PV size (KW)	DG wind size (KW)	Power loss (KW)	Reduction %
Base case	–	–	–	–	224	–
ZOA	61	64	445.7	266.07	45.74	79.58
WOA	65	60	456	304	53.16	76.27
GWO	18	64	405.12	304	74.28	66.84
GA	61	60	221.82	217.05	65.26	70.87

**Fig. 8:**
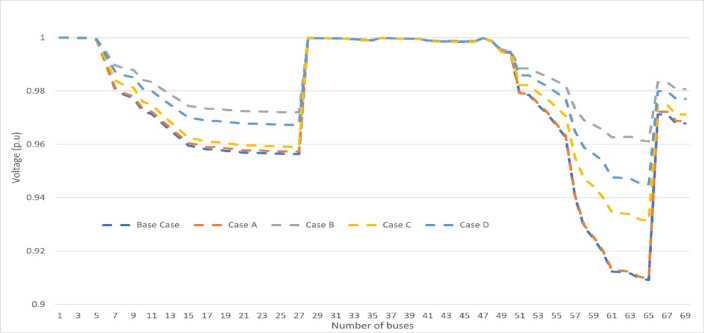
Voltage Profile for IEEE 69-bus radial for part (1) cases against the base case with applied ZOA.

The outcomes demonstrated that applying peak load shifting reduces power loss remarkably in comparison to case (C). The optimal location for integrating PV modules and wind modules along with EV on the network is at bus 61 and 60, respectively. Moreover, utilizing ZOA achieves the maximum power loss reduction of 79.58%, followed closely by WOA, GA, and GWO, respectively. Figure 8 represents the voltage profile using no DG as the base case against the integration of two DGs for cases from (A) to (D) with applying ZOA.

**Fig. 9:**
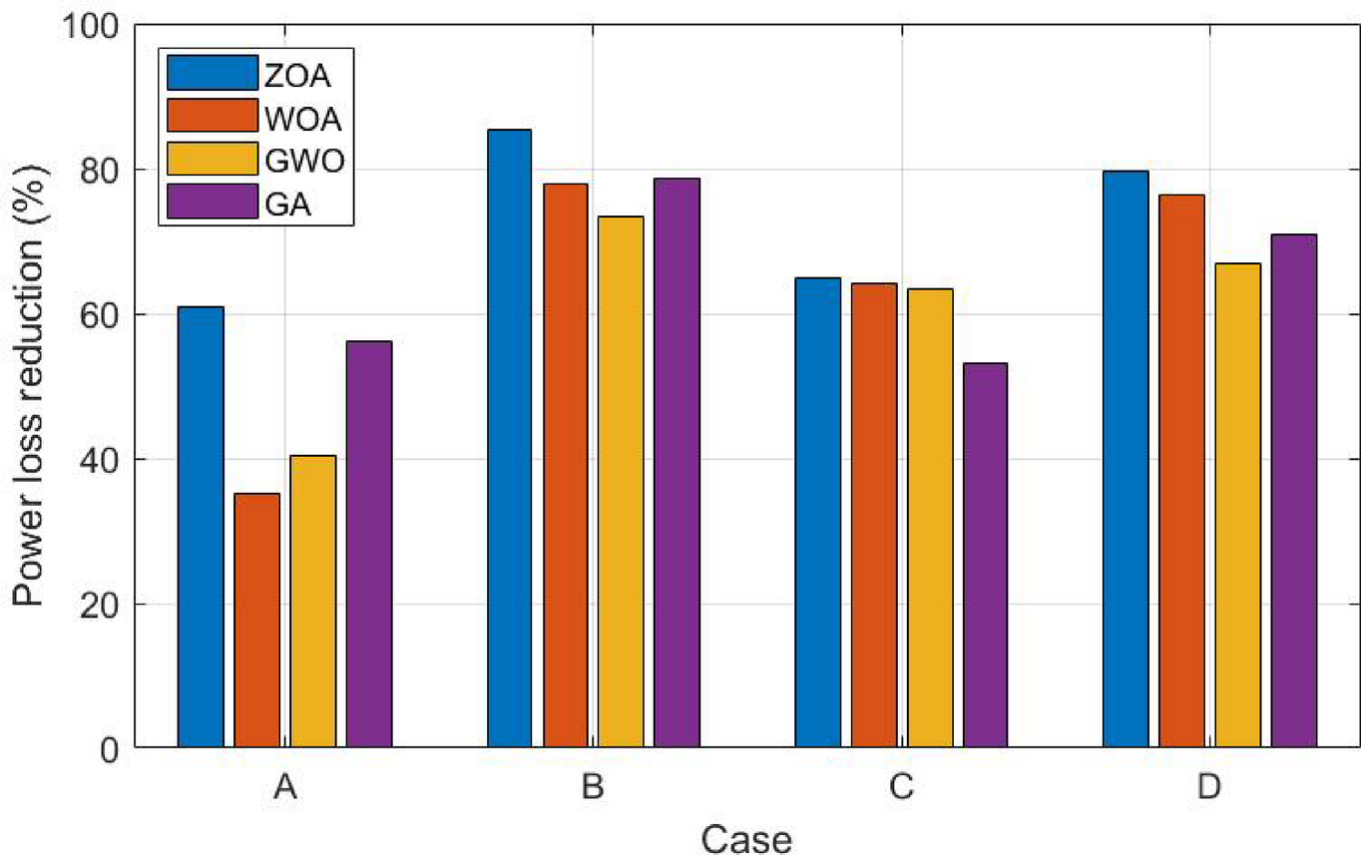
Part (1) comparison results for all cases.

The figure shows that integrating 2 DGs on the distribution system enhances the voltage profile. Also, integrating EV and applying peak load shifting with load variance in case (D) considering fixed integration of RER, improves the voltage profile significantly compared to cases (A) and (C). Figure 9 showcases a comparison between all cases for part (1) with respect to power loss reduction percentage.

**Fig. 10:**
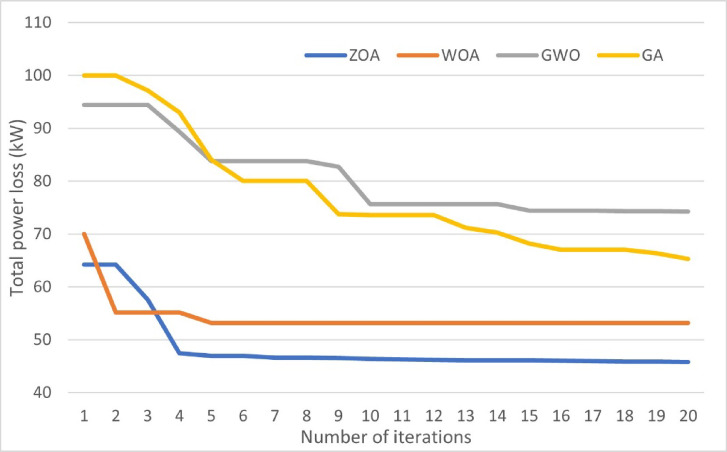
Conversions rates for ZOA, WOA, GWO and GA in case (D).

The figure illustrates that ZOA outperforms WOA, GWO, and GA in achieving the maximum power loss reduction. Also considering the load variance in case (B) and peak load shifting reflects an increased power loss reduction in comparison to constant 100% loading as in case (A) by approximately 40%. Even though integrating EVs increases power losses, in case (C) power loss reduction increased relative to case (A) for the ZOA by nearly 8%. Moreover, applying peak load shifting along with EV integration in case (D) increases power loss reduction by almost 23% compared to case (C). Figure 10 shows the conversion rates for the ZOA, WOA, GWO, and GA in case (D) for the first 20 iterations.

The curves indicate that the ZOA outperforms the WOA, GWO, and GA in reaching the optimal solution requiring fewer iterations and less time for simulation. Accordingly, the rest of the study has been simulated utilizing the ZOA only.

**Fig. 11:**
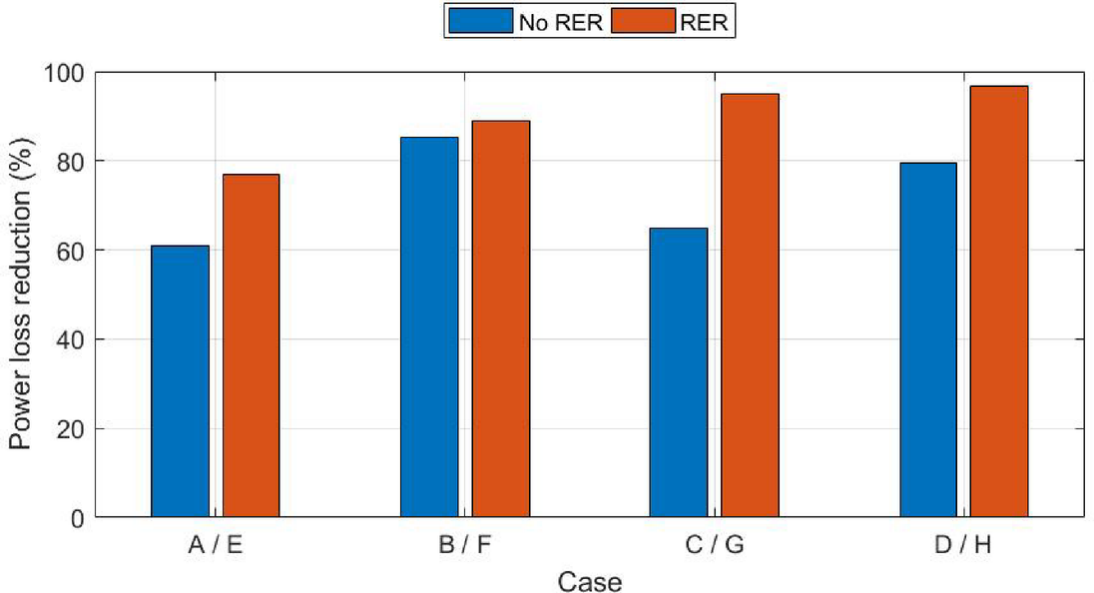
Part (1) and (2) comparison results for all cases using ZOA.

In part (2), the whole configuration has been assessed after considering the stochastic behavior of wind/PV. Except for cases (G) and (H), where the system has perceived the variable loading profile to evaluate the system under total uncertainties. The wind/PV were integrated as two DGs (hybrid) using the PDF of their forecasted data in winter over 24 h with a level of integration of 60% and 40%, respectively, of the total renewable generation capacity. The simulation was utilized by using the ZOA with 20 iterations. Table 7 summarizes the results of cases from (E) to (H) during the winter season for part (2). Figure 11 showcases a comparison between all cases for part (2) with respect to power loss reduction percentage by ZOA.

**Table 7:** Simulation cases for Part (2) in the winter season.

Applied Algorithm	Optimal location (1) PV	Optimal location (2) Wind	DG PV size (KW)	DG Wind size (KW)	Power loss (KW)	Reduction %
Base case	–	–	–	–	224	–
Case (E)	12	61	315.48	163.09	51.73	76.91
Case (F)	61	11	360.25	108.86	24.64	89.00
Case (G)	12	61	125.95	39.7	11.26	94.97
Case (H)	61	36	151.42	92.89	7.16	96.80

The figure indicates that case (H) which incorporates EV and RER during uncertainties and after applying peak load shifting reduces power loss the most among all cases. Despite connecting EV on the network without PLS increases power losses, case (C) and (G) power loss reduction increased in comparison to case (A) due to the V2G and G2V alleged scenarios.

**Fig. 12:**
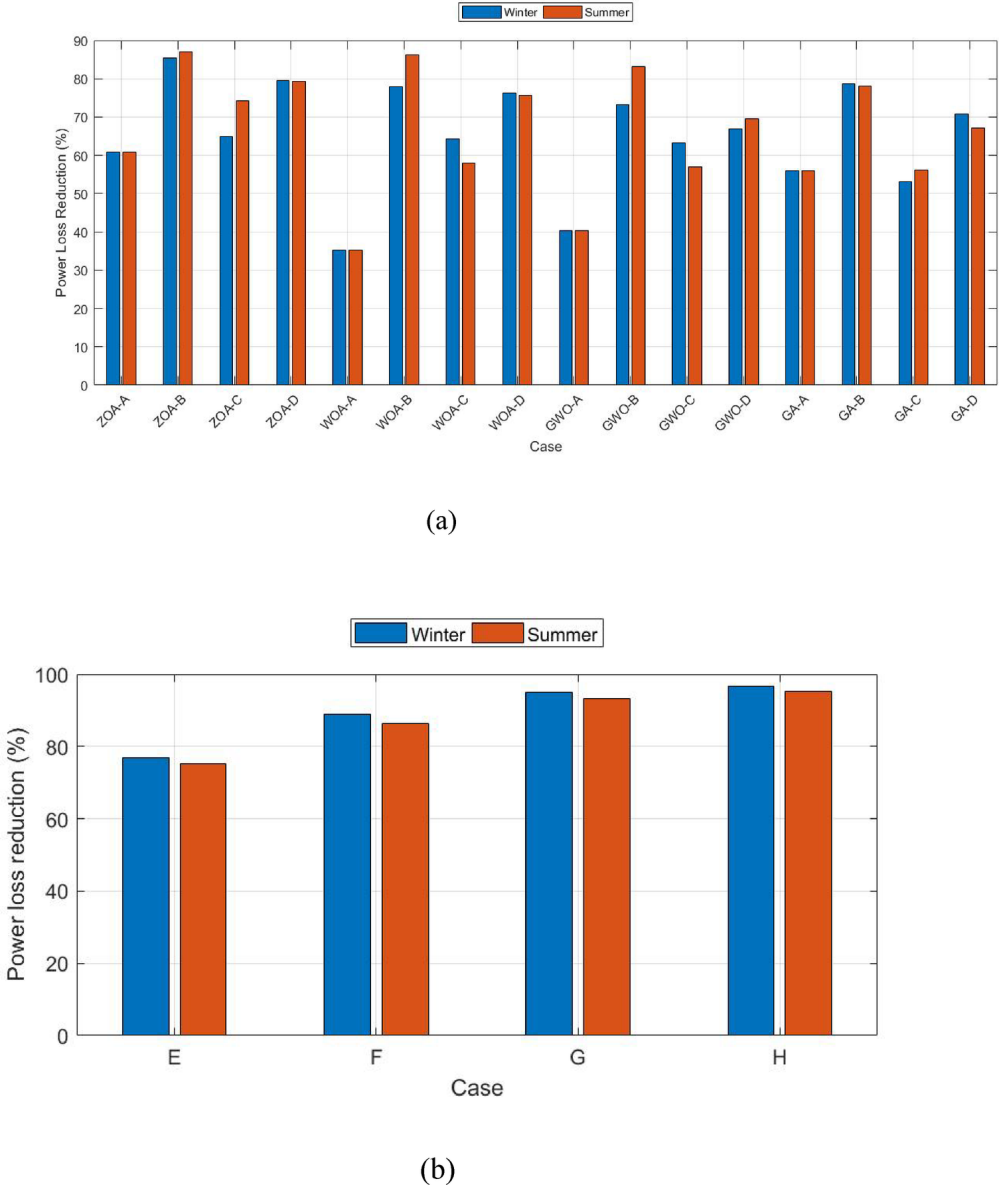
Comparison for all cases results for (**a**) Part (1) and (**b**) Part (2) (Summer versus Winter seasons).

Secondly, the whole configuration was repeated with the same sequence and steps with the integration of the summer season data. Table 8 summarizes all results for part (1) and part (2) during summer season cases from (A) to (H) and Fig. 12 shows case a comparison between all cases results for summer versus winter seasons for part (1) and part (2).

**Table 8:** Summary for all case studies during the summer season in (a) part (1) and (b) part (2).

Algorithm type	Case	Optimal location (1)	Optimal location (2)	DG PV size (KW)	DG Wind size (KW)	Power loss (KW)	Power loss reduction %
(a)							
ZOA	A	61	64	433.33	303.96	87.78	60.81
	B	61	64	454.48	302.98	28.87	87.11
	C	61	64	455.05	289.63	57.73	74.23
	D	61	64	443.24	296.39	46.11	79.42
WOA	A	65	63	30.04	245.84	145.23	35.17
	B	64	62	419.68	282.72	30.97	86.17
	C	65	27	401.4	264.94	93.98	58.04
	D	64	61	370.36	268.5	54.64	75.61
GWO	A	65	27	432.28	219.14	133.58	40.37
	B	63	62	427.26	298.87	37.44	83.29
	C	62	19	456	54.2	96.49	56.92
	D	63	64	456	195.78	68.06	69.62
GA	A	64	62	456	247.7	98.4	56.07
	B	61	62	292.74	218.77	48.94	78.15
	C	62	24	374.55	148.25	98.26	56.13
	D	64	21	415.42	196.61	73.44	67.21

(b)							
ZOA	E	30	61	186.34	98.92	55.6	75.18
	F	12	61	201.54	58.84	30.28	86.48
	G	8	2	253.07	11.01	15.23	93.20
	H	9	2	190.3	193.02	10.77	95.19

The tables and figures indicate that in part (1) ZOA power loss reduction for cases (B), (C), and (D) reached 87%, 74%, and 79%, respectively. While in part (2) the power loss reduction for cases (E), (F), (G), and (H) reached approximately 75%, 86%, 93%, and 95%, respectively.

## Discussion

This paper presented an energy management strategy combining the integration of EVs and peak load shifting to optimally utilize the size and location of RERs on the distribution network during winter and summer seasons. The applied DSM strategy implemented the ZOA in case of load, RESs, and EVs uncertainties. The first phase of the simulation started by processing the winter season data.

In part (1) the system investigated the ZOA performance in comparison to WOA, GWO, and GA across four cases while considering fixed RER integration. In part (2) the remaining four cases investigated the ZOA performance only taking into consideration the stochastic behavior of RERs.

In case (A), the investigation focuses on the system performance under constant output power generation for the RERs with 100% loading utilizing the four optimization algorithms. The results revealed that ZOA achieved the maximum power loss reduction of 60.81% followed by GA, GWO, and WOA with a power loss reduction of 56.07%, 40.37%, and 35.17%, respectively. While in case (B), the load uncertainty is considered with the implementation of peak load shifting. The results indicated an increase in the power loss reduction by applying ZOA, WOA, GWO, and GA by 85.36%, 77.85%, 73.28%, and 78.75%, respectively.

In case (C), the incorporation of EV is considered with 100% loading and fixed integration of RERs. Although EV integration increases power losses, results illustrated an increase in power loss reduction across the four algorithms compared to case (A). The power loss reduction for ZOA, WOA, GWO, and GA reached 64.81%, 64.22%, 63.34%, and 53.17% respectively. While applying peak load shifting in case (D), the power loss reduction increased to 79.58%, 76.27%, 66.84%, and 70.87% for ZOA, WOA, GWO, and GA, respectively. Even though the power losses decreased but the capacity of the utilized DG increased for the PV and wind during the optimization process by the ZOA. The approach optimally places the DGs closer to the load demand thus minimizing the resistive losses associated with long-distance power transmission. Furthermore, DG units support load demands improving the VSI and confirming that the marginal increase in size yields a proportionately significant reduction in losses and an overall enhancement in grid reliability.

In part (2), the ZOA is utilized to integrate the PDF of wind/PV. In case (E),100% loading is considered while in cases (F), (G), and (H) the load variance is taken into consideration. The power loss reduction for cases (E) and (F) reached 76.91% and 89%, respectively. Results also show that considering full system uncertainty reached the maximum power loss reduction of 94.7% in case (G) which is the worst-case scenario. Also, after applying the peak load shifting in case (H), the maximum power loss reduction is 96.8%. Case (G) should serve as a foundational basis and will be thoroughly utilized in conducting a more comprehensive investigation in the subsequent stage of the research. In the second phase of the simulation, the summer season data is processed. Results indicated that considering the uncertainties in load, EVs and RERs affect the power loss reduction in the winter season more than in summer season by approximately 5%. The usage and consumer behavior during which emphasis the network’s overall performance.

### Comparison with existing literature

In previously published research, a variety of strategies were applied to optimally select the size and location of DGs. Table 9 presents a comprehensive comparison between the proposed algorithm and previous research. The comparison encompasses several factors including the optimization algorithm, the integration of DGs and EVs, and the formulated objective function. Few prior studies combined the generation mix of DGs and EV on IEEE 69-bus system for power loss reduction. This research takes a more integrated comprehensive approach by simultaneously addressing both aspects within the same framework under various uncertainty conditions on large scale networks. Consequently, the proposed optimization technique is implemented for power loss reduction and applying DSM through peak load shifting for cost savings and system stability.

**Table 9:** Comparison between the proposed work and previous research.

No	Test system	DG	EV	DG + EV	Objective function	Proposed algorithm
1	IEEE 33-bus	√	X	X	Minimize daily active power losses and improve voltage profile	Particle Swarm Optimization (PSO) and Butterfly Optimization (BO)^51^
2	IEEE 33-bus and IEEE 69-bus	√	√	X	Lessen the active power losses, enhance VSI and adequate reliability	Hybrid grey wolf optimization and particle swarm optimization (HGWOPSO)^52^
3	IEEE 37-bus	√	√	√	Reducing total real power loss and improving system performance indices	GA^53^
4	IEEE 33-bus	√	√	√	Line loss reduction index, voltage profile improvement index and penetration level index	Dynamic fault tree analysis and Bayesian Optimization Technique^54^
5	IEEE 33-bus and IEEE 69-bus	√	√	√	Increase power loss reduction and VSI	Analytical method^55^
6	IEEE 69- bus	√	√	√	Minimize power loss and apply peak load shifting	Proposed work

### High EV penetration level

The cumulative charging demand of EVs on the power networks significantly impacts grid efficiency and stability. Effective optimal charging strategies assess in balancing these demands influenced by the penetration levels of EVs on the network. Accordingly, adaptive solutions shall be considered to scale with the EV demand based on the varying penetration levels. At low penetration levels, the limited number of EVs does not dramatically affect voltage profile and peak demand. On the other hand, the increased penetration levels reflect negatively on the power losses and voltage stability which highlights the need for optimal charging strategies^56^. Moreover, dynamic integrated solutions for infrastructure including electric vehicle charging stations (EVCS) are required to manage the high penetration levels^57^.

In light of the above, further analysis is implemented on the system to examine the impact of high penetration levels of PHEVs to optimally integrate EVCS on the proposed model before and after PLS aiming to flatten the demand curve, reduce costs, and improve grid efficiency.

The investigation is carried out on the IEEE 69-bus system which entails a fixed integration of wind/PV modules considering the uncertainty of EVs and load profile. Based on another simulation for case (G), the optimal DG integration of PV and wind is set at bus numbers 61 and 36 with a capacity of 200 kW and 55 kW, respectively. The optimal location and capacity of EVCS is optimized using the ZOA. Table 10 summarizes a comparison between different penetration levels of PHEV impact on power loss and voltage index with a focus on voltage at bus 61.

**Table 10:** Summary for four different penetration levels of EV.

PHEV penetration level (%)	Optimal EVCS location	Optimal EVCS capacity (kW, kVAR)	Power loss (kW)	Voltage index	Voltage at bus 61 (V)
Base Case	–	–	224.96	0.0266	0.9197
10%	22	357, 188	128.98	0.02	0.9550
15%	37	191, 354	127.87	0.0198	0.9568
20%	54	739, 308	126.377	0.0196	0.9579
30%	56	904, 561	125.39	0.0193	0.9618

The table indicates that high penetration levels of EVs along with DERs lead to a reduction in voltage index and an increase in power losses. However, it is noteworthy that at 20% and 30% EV integration levels on the network incorporating DERs, the voltage at bus 61 reached 0.9579 and 0.9618, respectively. The best possible plan for charging EVs aiming to minimize power loss and stabilize voltage levels is at 15%.

**Fig. 13:**
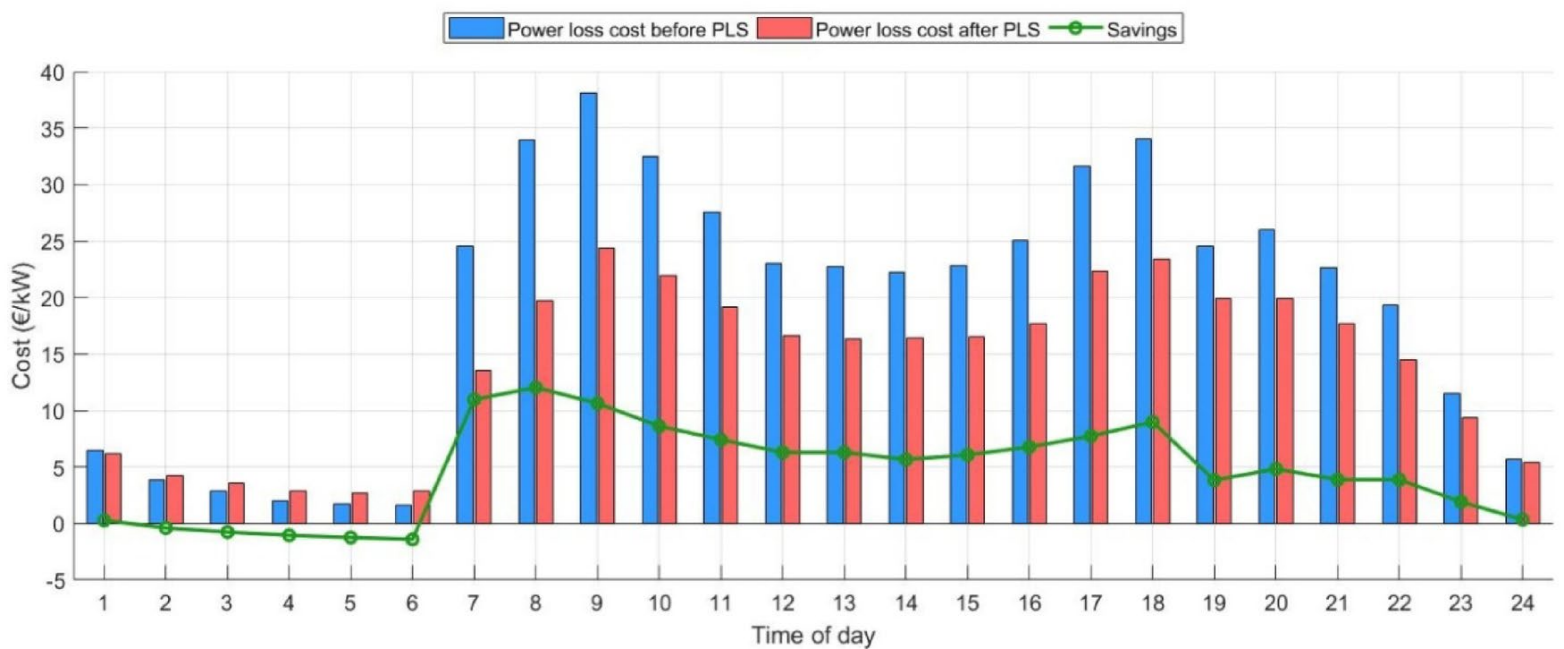
The hourly savings after applying PLS on IEEE 69-bus system.

Using the time of use (TOU) pricing scheme^58^ and the average day-ahead hourly price data in Spain^59^, the proposed model has the potential to significantly reduce costs while maintaining system stability. Extended analysis has been implemented after PLS with a 15% EV penetration level. The ZOA optimized the integration of EVCS, PV, and wind at bus numbers 56, 61, and 36 with a capacity of 452 kW, 200 kW, and 55 kW, respectively. Figure 13 summarizes the hourly savings for integrating a cumulative penetration level of 35% from the total network for EVs and DERs before and after PLS.

The results indicate that the most expensive hour during the day before PLS is 9 for 38 €/kWh. On the other hand, the new loadability profile derived from PLS reduced the cost of this time to 24 €/kWh. Also, the applied strategy reduces the daily average power loss by nearly 24%. Subsequently, the average savings arising from this DSM are up to 4.65 €/kWh.

### Comparison with large-scale optimization

**Fig. 14:**
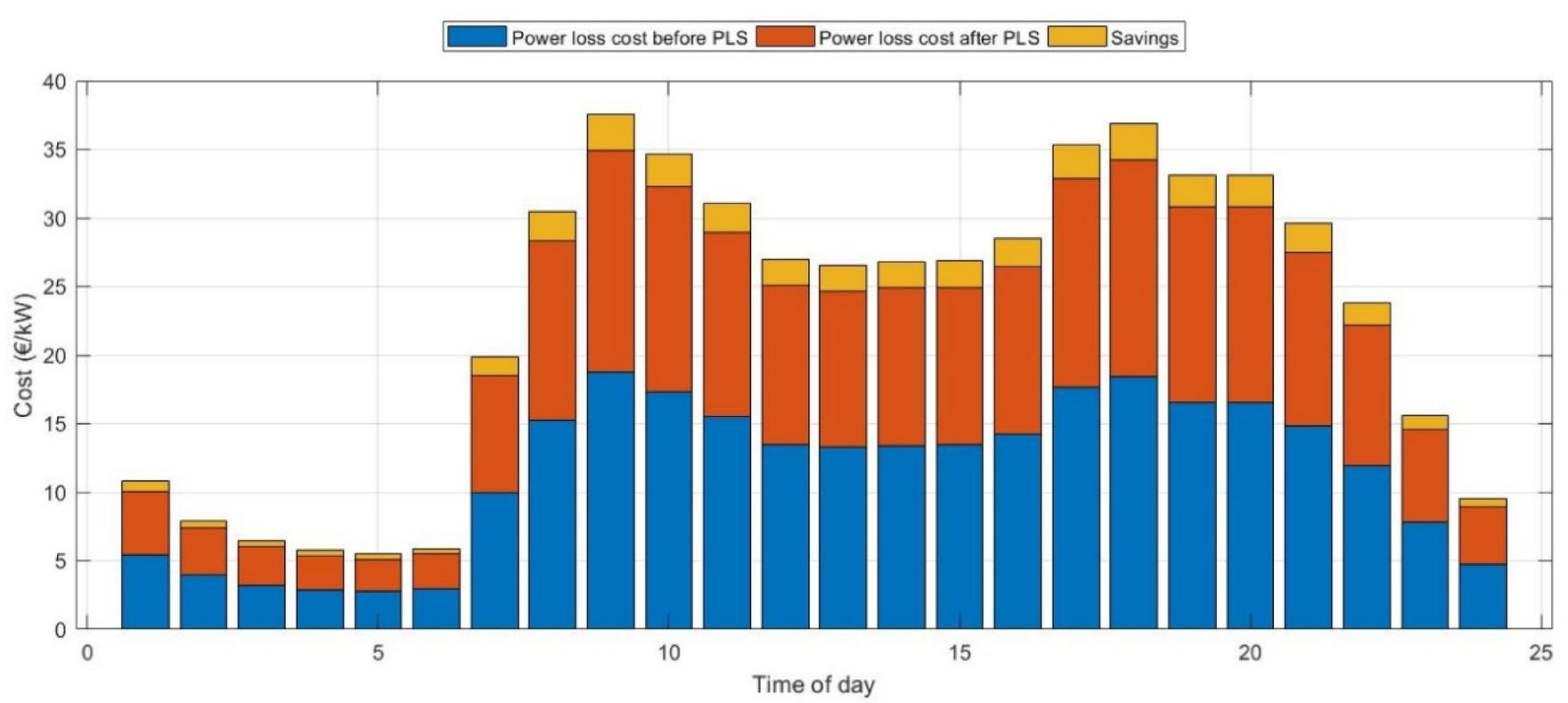
The hourly savings after applying PLS on IEEE 123-bus system.

The developed algorithm and implemented strategy have been tested on the IEEE 123-bus system^60^. The analysis has been conducted as in case (G) which is the worst-case scenario for the uncertainty of load, EVs, and DERs. The total active and reactive power loss is 3490 kW and 1920 kVAR, respectively with a voltage of 4.16 kV. Results revealed a reduction in power loss from 74.54 to 64.12 kW which is approximately 15%. The ZOA optimized the location of EVCS integration to be at bus number 39 with a capacity of 210.12 kW while considering PV and wind at bus numbers 67 and 21 with a capacity of 210.32 kW and 19.21 kW, respectively. Figure 14 summarizes the hourly savings for integrating a cumulative penetration level of 35% from the total network for EVs and DERs before and after PLS for IEEE 123-bus radial system.

The results indicate that the most expensive hours during the day before PLS are 9, 17 and 18 with a cost of 18.79 €/kWh, 17.67 €/kWh and 18.44 €/kWh, respectively. However, the new loadability profile derived from PLS reduced the cost of these hours to 16.13 €/kWh, 15.18 €/kWh and 15.82 €/kwh. Moreover, the new loadability profile derived from PLS reduces the daily average power loss by nearly 14%. Accordingly, the average savings from this DSM is up to 1.6 €/kWh.

## Conclusion

The growing integration of EVs and RERs as DGs into the power system holds great promise for reducing greenhouse gas emissions and improving system performance. But it also represents difficulties in maintaining efficiency and reliability. The dynamic charging pattern of EVs and the unpredictable nature of renewable energy contribute to power loss, voltage instability, and peak load surge. These issues underscore the need for effective DSM strategies such as shifting the peak load hours to off-peak hours which reduces costs and stress on the grid. To address these challenges, advanced optimization algorithms were developed to boost the overall system performance and reduce costs. One of the recent optimization techniques is the ZOA, inspired by the natural behavior of zebras and their zigzag patterns to defend predators thus finding the optimum solution. This paper presented an energy management strategy combining the integration of EVs and RERs to optimally utilize the size and allocation in smart cities, along with the demand-side management strategies. The proposed strategy was applied using the ZOA in case of load, RERs and EV uncertainties, and compared with WOA, GWO, and GA. The investigation simulated the proposed algorithm on a typical IEEE 69-bus and 123-bus system with multi-objective functions of power loss reduction and DSM by applying peak load shifting during winter and summer seasons. EV consumption is assumed to act as a load on the network for 12 h (G2V mode) and as a DG (V2G mode) for the rest of the day. The investigation for each season was applied in two parts, each of 4 cases. Part (1) considers a constant output power for the RER while part (2) considers the stochastic behavior of the RER. In cases (A) and (E), the integration is simulated using 100% loading throughout the day. While in cases (B) and (F) the load variance profile is considered after peak load shifting. And in cases (C) and (G) considered integration of the EV. Lastly, cases (D) and (H) considered all system uncertainty and applied peak load shifting. Results showed that the combined integration of DERs along with the EVs as a load and as a DG reduced power loss significantly with a remarkable effect on voltage profile. Moreover, applying the load peak shifting on the worst-case scenario decreased power losses the most. Furthermore, the ZOA performance with respect to achieving the minimum power loss and peak load shifting with optimal location and size of the RER, is better than the WOA, GWO, and GA in most cases. In addition, applying winter season data on the system reduced power loss in comparison to the summer season. Lastly, high penetration levels of EVs require optimal charging strategies to reduce expenses.

In future work, peak shaving shall be considered from the cost perspective from one hour to another. Also, other seasons should be considered using different machine-learning techniques to find the optimal location using ZOA and then compared to other techniques.

## Supplementary Information

Supplementary Information.

## Data Availability

The datasets used during the current study is available from the corresponding author on reasonable request.
