# Supplementary material for: Demand side management with electric vehicles and optimal renewable resources integration under system uncertainties
Source: Sci Rep. 2025 May 27;15:18570. doi: 10.1038/s41598-025-00752-6 (PMC12116743; doi:10.1038/s41598-025-00752-6)
Supplement: Supplementary file 1 — Supplementary Information. [file 41598_2025_752_MOESM1_ESM.docx]

# Appendix

# Appendix 1: IEEE 69-bus system data

| **Branch Number** | **From** | **To** | **R (Ω)** | **X (Ω)** | **P (kW)** | **Q (kVAR)** | **Branch Number** | **From** | **To** | **R (Ω)** | **X (Ω)** | **P (kW)** | **Q (kVAR)** |
| --- | --- | --- | --- | --- | --- | --- | --- | --- | --- | --- | --- | --- | --- |
| 1 | 1 | 2 | 0.0005 | 0.0012 | 0 | 0 | 35 | 3 | 36 | 0.0044 | 0.0108 | 26 | 18.55 |
| 2 | 2 | 3 | 0.0005 | 0.0012 | 0 | 0 | 36 | 36 | 37 | 0.064 | 0.1565 | 26 | 18.55 |
| 3 | 3 | 4 | 0.0015 | 0.0036 | 0 | 0 | 37 | 37 | 38 | 0.1053 | 0.123 | 0 | 0 |
| 4 | 4 | 5 | 0.0251 | 0.0294 | 0 | 0 | 38 | 38 | 39 | 0.0304 | 0.0355 | 24 | 17 |
| 5 | 5 | 6 | 0.366 | 0.1864 | 2.6 | 2.2 | 39 | 39 | 40 | 0.0018 | 0.0021 | 24 | 17 |
| 6 | 6 | 7 | 0.3811 | 0.1941 | 40.4 | 30 | 40 | 40 | 41 | 0.7283 | 0.8509 | 1.2 | 1 |
| 7 | 7 | 8 | 0.0922 | 0.047 | 75 | 54 | 41 | 41 | 42 | 0.31 | 0.3623 | 0 | 0 |
| 8 | 8 | 9 | 0.0493 | 0.0251 | 30 | 22 | 42 | 42 | 43 | 0.041 | 0.0478 | 6 | 4.3 |
| 9 | 9 | 10 | 0.819 | 0.2707 | 28 | 19 | 43 | 43 | 44 | 0.0092 | 0.0116 | 0 | 0 |
| 10 | 10 | 11 | 0.1872 | 0.0619 | 145 | 104 | 44 | 44 | 45 | 0.1089 | 0.1373 | 39.22 | 26.3 |
| 11 | 11 | 12 | 0.7114 | 0.2351 | 145 | 104 | 45 | 45 | 46 | 0.0009 | 0.0012 | 39.22 | 26.3 |
| 12 | 12 | 13 | 1.03 | 0.34 | 8 | 5 | 46 | 4 | 47 | 0.0034 | 0.0084 | 0 | 0 |
| 13 | 13 | 14 | 1.044 | 0.345 | 8 | 5.5 | 47 | 47 | 48 | 0.0851 | 0.2083 | 79 | 56.4 |
| 14 | 14 | 15 | 1.058 | 0.3496 | 0 | 0 | 48 | 48 | 49 | 0.2898 | 0.7091 | 384.7 | 274.5 |
| 15 | 15 | 16 | 0.1966 | 0.065 | 45.5 | 30 | 49 | 49 | 50 | 0.0822 | 0.2011 | 384.7 | 274.5 |
| 16 | 16 | 17 | 0.3744 | 0.1238 | 60 | 35 | 50 | 8 | 51 | 0.0928 | 0.0473 | 40.5 | 28.3 |
| 17 | 17 | 18 | 0.0047 | 0.0016 | 60 | 35 | 51 | 51 | 52 | 0.3319 | 0.1114 | 3.6 | 2.7 |
| 18 | 18 | 19 | 0.3276 | 0.1083 | 0 | 0 | 52 | 9 | 53 | 0.174 | 0.0886 | 4.35 | 3.5 |
| 19 | 19 | 20 | 0.2106 | 0.069 | 1 | 0.6 | 53 | 53 | 54 | 0.203 | 0.1034 | 26.4 | 19 |
| 20 | 20 | 21 | 0.3416 | 0.1129 | 114 | 81 | 54 | 54 | 55 | 0.2842 | 0.1447 | 24 | 17.2 |
| 21 | 21 | 22 | 0.014 | 0.0046 | 5 | 3.5 | 55 | 55 | 56 | 0.2813 | 0.1433 | 0 | 0 |
| 22 | 22 | 23 | 0.1591 | 0.0526 | 0 | 0 | 56 | 56 | 57 | 1.59 | 0.5337 | 0 | 0 |
| 23 | 23 | 24 | 0.3463 | 0.1145 | 28 | 20 | 57 | 57 | 58 | 0.7837 | 0.263 | 0 | 0 |
| 24 | 24 | 25 | 0.7488 | 0.247 | 0 | 0 | 58 | 58 | 59 | 0.3042 | 0.1006 | 100 | 70 |
| 25 | 25 | 26 | 0.3089 | 0.1021 | 14 | 10 | 59 | 59 | 60 | 0.3861 | 0.1172 | 0 | 0 |
| 26 | 26 | 27 | 0.1732 | 0.0572 | 14 | 10 | 60 | 60 | 61 | 0.5075 | 0.2585 | 1244 | 888 |
| 27 | 3 | 28 | 0.0044 | 0.0108 | 26 | 18.6 | 61 | 61 | 62 | 0.0974 | 0.0496 | 32 | 23 |
| 28 | 28 | 29 | 0.064 | 0.1565 | 26 | 18.6 | 62 | 62 | 63 | 0.145 | 0.0738 | 0 | 0 |
| 29 | 29 | 30 | 0.3978 | 0.1315 | 0 | 0 | 63 | 63 | 64 | 0.7105 | 0.3619 | 227 | 162 |
| 30 | 30 | 31 | 0.0702 | 0.0232 | 0 | 0 | 64 | 64 | 65 | 1.041 | 0.5302 | 59 | 42 |
| 31 | 31 | 32 | 0.351 | 0.116 | 0 | 0 | 65 | 11 | 66 | 0.2012 | 0.0611 | 18 | 13 |
| 32 | 32 | 33 | 0.839 | 0.2816 | 14 | 10 | 66 | 66 | 67 | 0.0047 | 0.0014 | 18 | 13 |
| 33 | 33 | 34 | 1.708 | 0.5646 | 19.5 | 14 | 67 | 12 | 68 | 0.7394 | 0.2444 | 28 | 20 |
| 34 | 34 | 35 | 1.474 | 0.4873 | 6 | 4 | 68 | 68 | 69 | 0.0047 | 0.0016 | 28 | 20 |

**Table A1.** Line and Bus Data of IEEE 69-bus network

# Appendix 2: Peak load shifting using mean and standard deviation

| **Time of Day** | **Previous load profile (kW)** | **Off-Peak hours** | **Peak Hours** | **New load profile (kW)** | |
| --- | --- | --- | --- | --- | --- |
| **1** | 833 | ✓ |  | | 895 |
| **2** | 784 | ✓ |  | | 845 |
| **3** | 746 | ✓ |  | | 808 |
| **4** | 734 | ✓ |  | | 795 |
| **5** | 734 | ✓ |  | | 795 |
| **6** | 746 | ✓ |  | | 808 |
| **7** | 921 | ✓ |  | | 982 |
| **8** | 1,070 |  |  | | 1,070 |
| **9** | 1,182 |  | ✓ | | 1,121 |
| **10** | 1,194 |  | ✓ | | 1,133 |
| **11** | 1,194 |  | ✓ | | 1,133 |
| **12** | 1,182 |  | ✓ | | 1,121 |
| **13** | 1,182 |  | ✓ | | 1,121 |
| **14** | 1,182 |  | ✓ | | 1,121 |
| **15** | 1,157 |  | ✓ | | 1,096 |
| **16** | 1,169 |  | ✓ | | 1,108 |
| **17** | 1,232 |  | ✓ | | 1,170 |
| **18** | 1,244 |  | ✓ | | 1,183 |
| **19** | 1,244 |  | ✓ | | 1,183 |
| **20** | 1,194 |  | ✓ | | 1,133 |
| **21** | 1,132 |  | ✓ | | 1,071 |
| **22** | 1,033 |  |  | | 1,033 |
| **23** | 908 | ✓ |  | | 969 |
| **24** | 784 | ✓ |  | | 845 |
| **Mean (kW)** | 1,033 |  |  | | 1,022 |
| **Std** | 195 |  |  | |  |
| **Minimum (kW)** | 935 |  |  | |  |
| **Maximum (kW)** | 1,130 |  |  | |  |
| **Total Diff (kW)** | 796.8 |  |  | |  |
| **Total Demand (kW)** | 24,780 |  |  | | 24,535 |
| **Peak Load (kW)** | 1,244 |  |  | | 1,170 |
| **Peak-to-average ratio** | 1.20 |  |  | | 1.14 |
| **Load Factor** | 0.83 |  |  | | 0.8736 |

**Table A2.** Calculation analysis for peak load shifting using mean and standard deviation for bus number 61

# Appendix 3: Peak load shifting using mean only

| **Time of Day** | **1** | **2** | **3** | **4** | **5** | **6** | **7** | **8** | **9** | **10** | **11** | **12** |
| --- | --- | --- | --- | --- | --- | --- | --- | --- | --- | --- | --- | --- |
| **Previous Load Demand** | 833 | 784 | 746 | 734 | 734 | 746 | 921 | 1,070 | 1,182 | 1,194 | 1,194 | 1,182 |
| **New Loadability** | 984 | 934 | 897 | 884 | 884 | 897 | 1,071 | 1,033 | 1,033 | 1,033 | 1,033 | 1,033 |
| **Time of Day** | **13** | **14** | **15** | **16** | **17** | **18** | **19** | **20** | **21** | **22** | **23** | **24** |
| **Previous Load Demand** | 1,182 | 1,182 | 1,157 | 1,169 | 1,232 | 1,244 | 1,244 | 1,194 | 1,132 | 1,033 | 908 | 784 |
| **New Loadability** | 1,033 | 1,033 | 1,033 | 1,033 | 1,033 | 1,033 | 1,033 | 1,033 | 1,033 | 1,033 | 1,058 | 934 |
| **Total Load Demand (Before / After shifting)** | | | | | 24,780 kW | | | | 24,030 kW | | | |
| **Mean demand (Before / After shifting)** | | | | | 1033 kW | | | | 1001 kW | | | |
| **Peak demand (Before / After shifting)** | | | | | 1244 kW | | | | 1071 kW | | | |
| **Load Factor (Before / After shifting)** | | | | | 0.83 | | | | 0.935 | | | |
| **Peak-to-average ratio (Before / After shifting)** | | | | | 1.20 | | | | 1.069 | | | |

**Table A3.** Calculation analysis for peak load shifting using mean only for bus number 61
